# Barcoded Pyrosequencing Reveals That Consumption of Galactooligosaccharides Results in a Highly Specific Bifidogenic Response in Humans

**DOI:** 10.1371/journal.pone.0025200

**Published:** 2011-09-26

**Authors:** Lauren M. G. Davis, Inés Martínez, Jens Walter, Caitlin Goin, Robert W. Hutkins

**Affiliations:** 1 Department of Food Science and Technology, University of Nebraska, Lincoln, Nebraska, United States of America; 2 School of Biological Sciences, University of Nebraska, Lincoln, Nebraska, United States of America; Duke-National University of Singapore Graduate Medical School, Singapore

## Abstract

Prebiotics are selectively fermented ingredients that allow specific changes in the gastrointestinal microbiota that confer health benefits to the host. However, the effects of prebiotics on the human gut microbiota are incomplete as most studies have relied on methods that fail to cover the breadth of the bacterial community. The goal of this research was to use high throughput multiplex community sequencing of 16S rDNA tags to gain a community wide perspective of the impact of prebiotic galactooligosaccharide (GOS) on the fecal microbiota of healthy human subjects. Fecal samples from eighteen healthy adults were previously obtained during a feeding trial in which each subject consumed a GOS-containing product for twelve weeks, with four increasing dosages (0, 2.5, 5, and 10 gram) of GOS. Multiplex sequencing of the 16S rDNA tags revealed that GOS induced significant compositional alterations in the fecal microbiota, principally by increasing the abundance of organisms within the Actinobacteria. Specifically, several distinct lineages of *Bifidobacterium* were enriched. Consumption of GOS led to five- to ten-fold increases in bifidobacteria in half of the subjects. Increases in Firmicutes were also observed, however, these changes were detectable in only a few individuals. The enrichment of bifidobacteria was generally at the expense of one group of bacteria, the *Bacteroides*. The responses to GOS and the magnitude of the response varied between individuals, were reversible, and were in accordance with dosage. The bifidobacteria were the only bacteria that were consistently and significantly enriched by GOS, although this substrate supported the growth of diverse colonic bacteria in mono-culture experiments. These results suggest that GOS can be used to enrich bifidobacteria in the human gastrointestinal tract with remarkable specificity, and that the bifidogenic properties of GOS that occur *in vivo* are caused by selective fermentation as well as by competitive interactions within the intestinal environment.

## Introduction

It has become increasingly recognized that the gastrointestinal microbiota plays a critical role in human health [1], [2], affecting nutrient utilization and adsorption by the host, the development and maturation of the immune system, and resistance to infections [3], [4], [5], [6]. Aberrations in the gut microbiota have been linked to several complex diseases, including inflammatory bowel disease [4], [7], [8], [9], obesity, atherosclerosis and cardiovascular disease [10], [11], [12], [13], type 2 diabetes [14], colorectal cancer [15], [16], [17], arthritis [18], and allergic diseases [4], [19]. Moreover, the discovery that it is possible to induce changes in the intestinal microbiota by dietary strategies [20], [21] has led to the suggestion that these aberrations or imbalances can be corrected and host health improved [22], [23].

One strategy by which the composition and metabolic activity of the intestinal microbiota can be modulated is via the introduction of prebiotics into the diet. Prebiotics are defined as “selectively fermented ingredients that allow specific changes, both in the composition and/or activity in the gastrointestinal microbiota that confer benefits upon host well-being and health” [24]. Several prebiotics are now widely used commercially in foods, including inulin, fructooligosaccharides (FOS), resistant starch, and galactooligosaccharides (GOS). There is now convincing *in vivo* evidence that shows that prebiotics can promote growth of bifidobacteria in the intestinal tract of infants and adults [25], [26]. For GOS in particular, 2 to 3 log increases in the number of bifidobacteria in fecal samples obtained from individual adults have been reported [27]. However, the human gut microbiota is composed of hundreds of species [22], and the impact of prebiotics on other members of the intestinal microbiota, especially those that remain unculturable, is generally less well understood.

The specificity of prebiotic substrates has been attributed to their selective fermentation in the intestinal tract by bifidobacteria and lactobacilli [25]. Indeed, genes encoding for pathways involved in metabolism of several oligosaccharides have been reported to be present in species of *Bifidobacterium* and *Lactobacillus* [28], [29], [30], [31], [32], [33]. Interestingly, however, in mono-culture, other colonic bacteria have also been reported to use prebiotics as an energy or carbon source, including species of *Clostridium*, *Enterococcus*, *Bacteroides*, and *Escherichia* [25]. In addition, hundreds of bacterial species colonize the human gastrointestinal tract, many of which are not culturable, and knowledge about their ability to utilize prebiotic substrates is currently very restricted.

Until recently, studies on the *in vivo* specificity of prebiotics have relied on either cultural enumeration methods that fail to detect the majority of microbial species present in the human gut [20], [34], [35], [36] or on molecular methods, such as quantitative real time (qRT)-PCR or fluorescent in situ hybridization, that are restricted to selected bacterial groups. Other methods, such as denaturing gradient gel electrophoresis (DGGE) and terminal-restriction fragment length polymorphism, can potentially detect alterations of any member within the bacterial population, but have a narrow dynamic range and only detect the most dominant species present. Despite these limitations, several studies have shown that the prebiotic response was not completely restricted to bifidobacteria. For example, Tannock and co-workers showed that FOS increased staining intensities of not only *Bifidobacterium adolescentis* but also *Collinsella aerofaciens* [37]. In a study using mice, Apajalahti and colleagues reported that inulin induced community shifts included increases of bifidobacteria and a decrease in clostridia, but the major changes were observed within previously unknown taxa [38]. Therefore, although the bifidogenic effect of most prebiotic carbohydrates is clearly established, knowledge about the effect on the entire community is still scarce.

Massive parallel sequencing of amplified 16S DNA tags via pyrosequencing now provides the means to quantify the fecal microbiota at increased depth and to span the entire microbial community. Thus, a much more detailed analysis of how prebiotics affect the microbiota can be achieved using this technique, and community wide shifts throughout the entire phylogenetic spectrum of the bacterial population can be measured. We recently reported that GOS, incorporated into caramel-like confections, increased the population of bifidobacteria when consumed by healthy adults at doses above 5 g per day, as assessed by cultural techniques, qRT-PCR, and DGGE [27]. However, other changes in the microbiota were less apparent, due to the limitations of these techniques. The goal of this current study, therefore, was to gain a more comprehensive perspective of the impact of GOS on the entire bacterial community in the fecal samples of these subjects using high throughput multiplex community sequencing of 16S rDNA tags. We discovered that GOS was remarkable for its ability to enrich specifically for bifidobacteria, despite the observation that the substrate is utilized by other colonic bacteria when assessed *in vitro*.

## Materials and Methods

### Experimental design

The study was approved by the Institutional Review Board at the University of Nebraska (IRB Approval Number: 2009019551EP), and written informed consent was obtained from all subjects. The details for the study design were previously described [27]. Briefly, caramel chews were administered to 18 healthy human volunteers during a 16 week period. The first two weeks were established as the baseline period, followed by four sequential testing periods during which chews were administered for three weeks with GOS dosages at levels of 0.0 g, 2.5 g, 5.0 g, and 10.0 g GOS per day. A final two-week washout period was performed at the end of the fourth testing period. Fecal samples were obtained weekly, and DNA was isolated using a method that includes both an enzymatic and mechanic cell lysis [27].

### Analysis of the fecal microbiota by pyrosequencing

Pyrosequencing of 16S rDNA tags was performed from fecal DNA as described previously [39]. Briefly, the V1-V3 region of the 16S rDNA gene was amplified by PCR from fecal DNA using primers adapted for the Roche-454 Titanium kit. A mixture (4∶1) of the primers B-8FM

(5′-CCTATCCCCTGTGTGCCTTGGCAGTCTCAGAGAGTTTGATCMTGGCTCAG-3′) and B-8FMBifido

(5′-*CCTATCCCCTGTGTGCCTTGGCAGTCTCAG*AGGGTTCGATTCTGGCTCAG-3′), were used as the forward primers. The primer A518R

(5′-*CCATCTCATCCCTGCGTGTCTCCGACTCAG*BBBBBBBBATTACCGCGGCTGCTGG-3′) containing an 8-base barcode sequence was used as the reverse primer. Sequences were then assigned to their respective samples via the barcode. The 8FMBifido was used in combination with primer 8FM, as 16S DNA sequences within the genus *Bifidobacterium* are not well amplified by the latter primer [40].

Equal amounts of the PCR products were combined and gel purified and then sequenced with the 454/Roche A sequencing primer kit using a Roche Genome Sequencer GS-FLX. Sequences were binned according to barcodes, using the Ribosomal Database Project (RDP) Pyrosequencing Pipeline (http://pyro.cme.msu.edu/) ‘Initial Process’ tool [41]. Default parameters were established to remove sequences containing any ambiguous nucleotides, except for the minimum sequences length, which was set to 300 bp. BioEdit Software was used to trim the quality approved sequences to 450 bp before submission to the sequence analyses (see below).

### Sequence analyses to characterize microbial populations

Sequences obtained by pyrosequencing were analyzed using taxonomy-dependent and taxonomy-independent approaches. First, the Classifier tool of the RDP was applied (with a minimum bootstrap value of 80%) to obtain a taxonomic assignment of all sequences. The Classifier approach allowed a fast determination of the proportions of bacterial groups at different taxonomic levels (phylum to genus). Alternatively, the sequences were assigned to Operational Taxonomic Units (OTUs). Accordingly, all sequences from each subject were individually aligned using the RDP Aligner web tool, and then clustered using the RDP Complete Linkage Clustering web tool (with a maximum distance cutoff of 97%; [41]). The OTU picking was performed on a per subject basis, as the entire data set from all of the subjects contained too many sequences for a quality alignment. OTUs that contained less than three sequences were excluded from the analyses. Using Statistical Analysis Software (SAS) to perform ANOVA, the OTUs that were significantly affected by the treatments in each subject were identified.

Representative sequences from each OTU whose abundance was significantly influenced by GOS were subjected to taxonomic classification using SeqMatch, an RDP web tool. From each statistically significant OTU identified, five random representative sequences were aligned to form consensus sequences using SeqMan Software (DNASTAR Lasergene). The consensus sequences were grouped and aligned according to phylum (Actinobacteria, Bacteroidetes, Firmicutes, Fusobacteria, Proteobacteria, and Verrucomicrobia), together with the most closely related type strains or entry in the NCBI database using Muscle 3.6 [42]. Phylogenetic trees were assembled by neighbor-joining with 1,000 bootstrap replicates with MEGA 4.0 Software [43]. Using visual analyses and a distance matrix, OTUs were assigned as sequence clusters with >97% identity, and consensus sequences were generated for each of the OTU sequence clusters, as described above.

Quantification of each OTU in each sample was performed by BLASTn analysis with a local database including all the quality controlled sequences generated by pyrosequencing. A BLASTn algorithm was used with a 97% cutoff (min. length 300 bp) to quantify each OTU within each sample. The OTUs that were closely related to *Bifidobacterium adolescentis* were quantified by BLASTn using a cutoff of 98% (min. length 300 bp) as clearly differentiated clusters could be identified that showed overlap with 97%. The quantification of OTUs in all subjects was then verified to ensure that individual sequences were not being assigned to different OTUs. In three occasions, OTUs that were initially identified as distinct had very high sequence similarities, and were thus merged together as single OTUs.

### Determination of community diversity

Two different methods, the generation of rarefaction curves and Shannon's index, were applied to determine the diversity of the fecal microbiota using 16S rDNA sequence data. The DNA sequences within each sample were aligned and clustered using RDP web tools Aligner and Complete Linkage Clustering. Individual cluster files corresponding to each fecal sample were used to construct Rarefaction curves and determine the Shannon's Index.

### Statistical analysis

To identify differences in the composition of the fecal microbiota induced through dietary treatments (0.0 g, 2.5 g, 5.0 g, and 10.0 g GOS) in all eighteen subjects, one-way ANOVA tests with repeated measures were performed. Samples obtained during the baseline and washout periods were not included within the statistical analysis. Post hoc pair-wise comparisons were done using Tukey's method. P-values of <0.05 were considered significant unless otherwise stated.

### *In vitro* utilization of GOS by bifidobacteria and other colonic bacteria

A total of 39 strains of bifiodbacteria were screened for their ability to use GOS as a growth substrate. Included were 19 lab strains (from ATCC, commercial sources, and the Department of Food Science Culture Collection) and 20 isolates obtained from subjects in the previous study [27]. Strains were grown anaerobically at 37°C in MRS broth containing 2% GOS (GTC Nutrition, Golden CO). Because the latter material contains 92% GOS, with the balance as lactose, glucose, and galactose, control cultures were prepared that contained an equivalent amount of these sugars (i.e., 0.16% final concentration). In addition, twenty-two anaerobic bacteria that were mainly of intestinal origin were also screened for their ability to use GOS as a growth substrate. All bacteria were obtained from the USDA ARS Culture Collection (Peoria, IL) and included strains of *Bacteroides thetaiotamicron*, *Bacteroides distasonis*, *Bacteroides fragilis*, *Bacteroides uniformis*, *Bacteroides ovatus*, *Clostridium butyricum*, *Clostridium histolyticum*, *Clostridium bifermentans*, *Clostridium difficile*, *Clostridium innocuum*, *Clostridium paraputrificum*, *Clostridium perfringens*, *Clostridium ramosum*, *Clostridium rumen*, *Clostridium sporogenes*, *Enterococcus faecalis*, *Enterococcus faecium*, *Enterobacter aerogenes*, and *Streptococcus salivarius*. Bacteria were initially propagated in Brain Heart Infusion (BHI) or Reinforced Clostridial Agar (RCA). To assess growth on GOS, cells were transferred (2%) into a basal medium [5 g/L Peptone No 3 (Becton, Dickinson, and Company), 5.0 g/L Casitone (Becton, Dickinson, and Company), 0.5 g/L L-Cysteine (Sigma), 40 mL Salt Solution, 10 mL Hemin (Sigma), 900 µL Vitamin K_3_ (Sigma), and 1 g/L Yeast Extract (Becton, Dickinson, and Company)] containing 1% GOS (GTC Nutrition, Golden, CO). Control cultures containing 0.08% mono- and disaccharides were prepared as above.

All cultures were incubated at 37°C in an anaerobic chamber (Forma Scientific, Marietta, Ohio) containing an atmosphere of 85% nitrogen, 10% hydrogen, and 5% carbon dioxide and assessed for growth by optical density measurement at 600 nm in a Beckman Model 640 spectrophotometer. Each experiment was done in triplicate and the average optical densities were determined.

## Results

### The effect of GOS on the fecal microbial communities

A total of 288 fecal samples were included in this study. Pyrosequencing resulted in a total of 2.3 million sequences. After quality control analysis (see Methods), an average of 8,200 sequences per sample was obtained. The mean sequence length was approximately 450 bp. An average of 2,022 OTUs was identified per subject. To assess the effect of GOS on the bacterial diversity in fecal samples, rarefaction curves for all eighteen subjects were generated (data not shown), and Shannon's diversity indices were calculated. This analysis revealed that consumption of GOS did not alter bacterial diversity of the fecal samples (p<0.0713).

The overall composition of the gut microbiota in the 18 individuals included in this study is in general agreement with that of previous studies [13]. During the baseline period (no dietary modulation), the microbiota was dominated by two phyla, Firmicutes (64%) and Bacteroidetes (28%). Other phyla detected included Actinobacteria (3%), Verrucomicrobia (1%), and Proteobacteria (1%). Approximately 3% of the sequences remained unclassified. At the family level, the predominant groups were the Lachnospiraceae (31%), Ruminococcaceae (18%), Bacteroidaceae (12%), and Bifidobacteriaceae (2%). The most common genera included *Bacteroides* (12.2%), *Fecalibacterium* (7.7%), *Blautia* (7.4%), *Ruminococcus* (3.7%), *Roseburia* (2.2%), *Bifidobacterium* (1.5%), and *Dorea* (1.3%).

Sequence proportions determined by pyrosequencing were used to determine the effect of GOS on the composition of the gastrointestinal microbiota. The groups that were significantly affected are shown in Table 1, according to phylum, family, genus (by RDP Classifier), and OTUs. The control treatment (0.0 g GOS in confections) had no effect on the fecal microbiota, as the microbial populations during this period were not significantly different from those during the baseline and washout periods (although slight increases in the family Bacteroidaceae and the genus *Bacteroides* were detected). In addition, no significant changes in the fecal microbiota were detected for a dose of 2.5 g GOS. In contrast, consumption of 5.0 g GOS led to several significant changes. There were significant increases (p<0.05) in the family Bifidobacteriaceae and the genus *Bifidobacterium*, compared to the control dose. At the species level, the abundance of only one OTU, corresponding to the species, *Fecalibacterium prausnitzii*, increased significantly at this dose. In contrast, significant decreases in abundance were observed at both the family and genus levels for *Bacteroidaceae* (p<0.01) and *Bacteroides* (p<0.01), respectively, at the 5.0 g dose compared to the control.

**Table 1:** Abundance of bacterial taxa affected by GOS consumption in fecal samples of eighteen human subjects as determined by pyrosequencing of 16S rRNA tags.

Proportion of bacterial taxa expressed in percentage (Mean ± SD)							
	Baseline 1	0.0 g 2	2.5 g 2	5.0 g 2	10.0 g 2	Washout 1	P value 3
Phylum							
Actinobaceria	2.52±2.34	2.58±3.59	3.69±4.33	5.39±6.11	7.19±8.88	2.09±2.51	<0.0001
Family							
Bfidobacteriaceae	1.56±2.14	1.69±2.65	2.50±3.43	4.27±5.18	6.14±7.08*** §§	1.24±2.10	<0.0001
Bacteroidaceae	12.22±7.43	15.03±10.66	13.29±9.24	11.20±9.11**	11.66±9.22**	13.69±8.27	0.0030
Genus							
*Bifidobacterium*	1.28±1.81	1.40±2.20	2.13±2.99	3.61±4.46	5.20±6.18*** §§	1.05±1.82	0.0002
*Bacteroides*	12.22±7.43	15.03±10.66	13.29±9.24	11.20±9.11**	11.66±9.22**	13.69±8.27	<0.0001
Species (OTUs)							
*Bifidobacterium adolescentis*	0.37±0.56	0.34±0.89	0.46±0.86	0.85±1.09	1.03±1.55*	0.21±0.48	0.0101
*Bifidobacterium* spp I	0.15±0.36	0.18±0.33	0.25±0.55	0.52±1.13	0.77±1.41* §	0.12±0.25	<0.0001
*Bifidobacterium* spp II	0.46±0.94	0.60±1.53	0.76±1.72	1.41±2.38	2.00±3.45* §	0.22±0.45	<0.0001
*Bifidobacterium* spp III	0.62±1.21	0.78±2.19	0.98±2.02	1.82±3.30	2.50±4.55* §	0.40±0.92	0.0088
*Bifidobacterium longum*	0.09±0.23	0.09±0.23	0.12±0.32	0.22±0.50	0.33±0.85*	0.15±0.38	0.0232
*Bifidobacterium catenulatum*	0.15±0.34	0.27±0.88	0.56±1.38	0.51±1.16	0.91±2.08**	0.28±0.78	0.0105
*Faecalibacterium prausnitzii*	3.52±2.71	3.21±2.26	3.71±2.67	4.37±3.67*	3.16±1.82†	3.42±2.28	<0.0001
*Coprococcus comes*	2.90±2.04	2.40±1.75	2.12±1.24	1.99±1.55	1.78±1.11*	2.15±1.30	<0.0001

1 Bacteria populations are averages of the two time points of the baseline period and the two time points of the washout 2 period.

2 Bacteria populations are averages of all three time points of the feeding periods.

3 Bacterial populations during the dietary treatments were compared to eachother with repeated measures ANOVA and Tukey's post hoc test.

Significantly different to 0.0 g:

*(p<0.05),

**(p<0.01),

***(p<0.001).

Significantly different to 2.5 g:

§ (p<0.05),

§§ (p<0.01).

Significantly different to 5.0 g:

† (p<0.05).

At the 10.0 g GOS dose, additional differences in the proportions of several phyla (using taxonomy-based analysis) were observed (Table 1). There was a significant increase in Actinobacteria compared to the control (p<0.001), as well as compared to the 2.5 g dose (p<0.05). This change was associated with an increase both in the family Bifidobacteriaceae, the genus *Bifidobacterium*, and several OTUs related to *Bifidobacterium* species. Although there were not significant differences between the 5 gram and 10 gram dose in Bifidobacteriaceae, the genus *Bifidobacterium*, and *Bifidobacterium* species, the amount of bifidobacteria at 10 gram GOS was consistently higher than at 5 gram. In addition, bifidobacteria were significantly increased at 10 gram GOS when compared to the 2.5 gram dose (Table 1). Collectively, the abundances of bifidobacteria determined by pyrosequencing were highly correlated (*r* = 0.7629, *p*<0.0001) with the cell counts previously obtained by qRT-PCR [27] (Figure S1). This supports previous findings that show that our pyrosequencing approach allows a quantitative determination of bifidobacteria in human fecal samples.

There were few bacterial taxa other than bifidobacteria that were influenced by GOS, based on a taxonomy-based analysis (Table 1). Statistically significant decreases were observed only within the family Bacteroidaceae (p<0.05) and the genus *Bacteroides* (p<0.05) when compared to the control dose of GOS. In contrast, the OTU-based approach identified two additional taxa, *Coprococcus comes* and *F. prausnitzii*, whose abundances differed significantly at 5 and 10 g doses. However, no trend was apparent from these results (Table 1). Although few taxa were identified that significantly decreased with the administration of GOS when all 18 subjects were assessed collectively, our analysis nonetheless showed that different bacterial lineages were decreased in individual subjects. As shown in Figure S2, the changes were detected in a small number of subjects and occurred primarily within taxonomically diverse members within the phyla Firmicutes (Figure S2A) and Bacteroidetes (Figure S2B). Most of these taxa were reduced by GOS, but no consistent pattern was detected among subjects. Thus, it appears that although GOS induces a rather selective increase of different lineages of bifidobacteria, GOS does not result in a consistent increase of another bacterial group or a significant decrease of particular bacterial groups.

### GOS enhances different lineages of bifidobacteria

**Figure 1:**
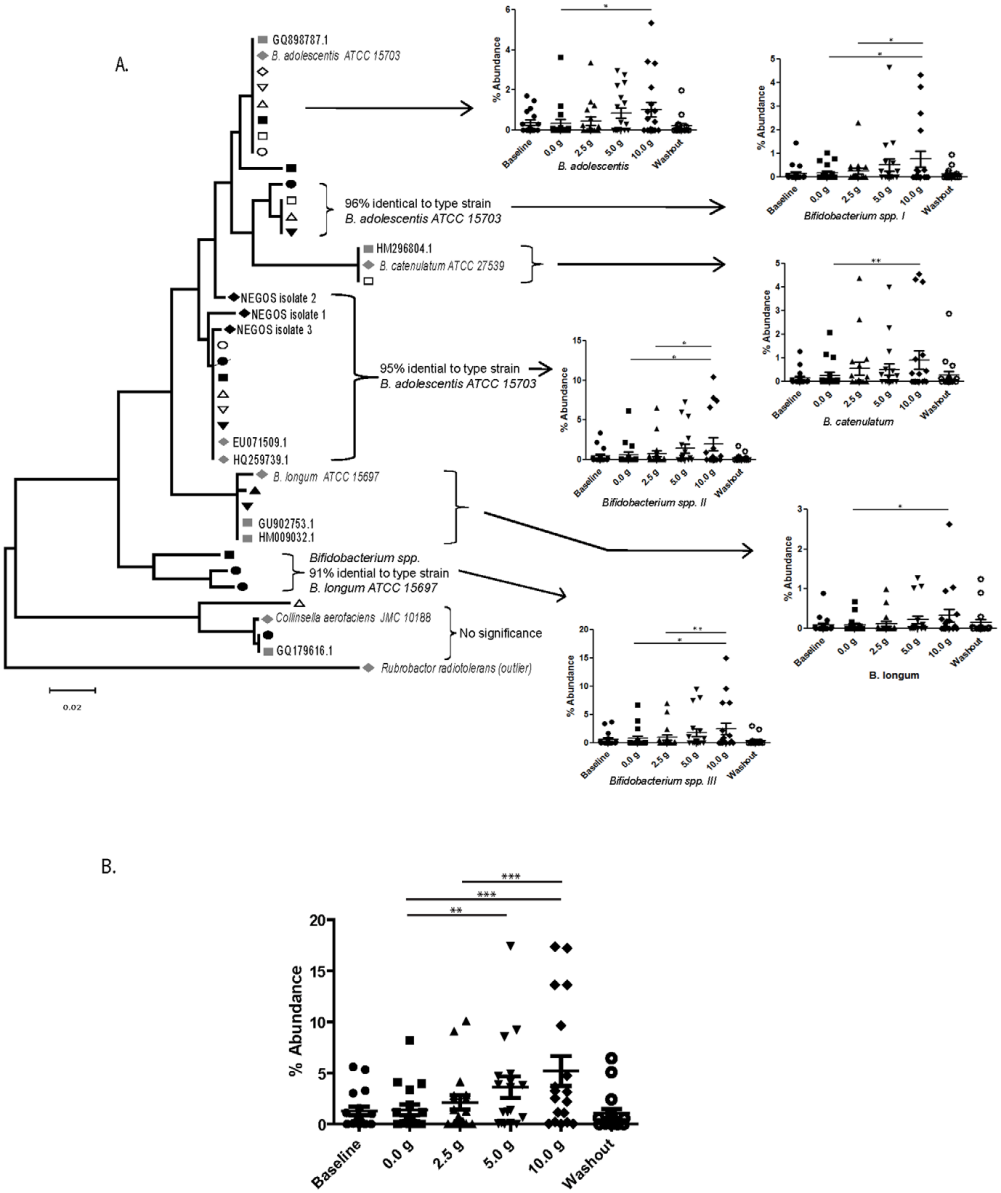
Characterization of the fecal microbiota in eighteen subjects that consumed increasing doses of GOS by multiplex pyrosequencing of 16S rDNA tags. A phylogenetic tree that encompasses the phylum Actinobacteria is shown (**A**). The tree contains representative sequences of all OTUs detected that were significantly affected by GOS in individual subjects together with sequences of related entries in the database. The latter includes both type strains of known species and sequences from molecular studies of human fecal samples. Sequences were aligned using Muscle 3.6 and the trees were constructed using the neighbor-joining algorithm with 1,000 bootstrap replicates in MEGA 4.0. The sequences from individual subjects are labeled using open black and closed black symbols, and type strains and other sequenced human strains are indicated by grey symbols. Those OTUs that were not significantly affected in all eighteen subjects were labeled as “No significance”. Graphs to the right of the trees show the abundance of the OTUs and bacterial groups that were significantly affected by GOS. The abundances of all of the *Bifidobacterium* species affected by GOS consumption, for all eighteen subjects, are shown in **B**. These graphs show mean proportions of the three individual samples taken during the treatment periods for each subject. Baseline and washout refer to samples taken in periods where no GOS was consumed. Repeated measures ANOVA in combination with a Tukey's post-hoc test were performed to indentify differences between treatment and control periods, where * = p<0.05, ** = p<0.01, and *** = p<0.001. Baseline and washout periods were not included in the statistic analysis.

A BLASTn analysis revealed that eight OTUs had statistically significant changes in abundance at the 10 g GOS dose, six of which were assigned to the genus *Bifidobacterium*. Three of the OTUs showed a high similarity (>97%) to described *Bifidobacterium* species, *B. adolescentis*, *B. longum*, and *B. catenulatum* (Table 1, Figure 1A). The other OTUs (*Bifidobacterium* spp I, II, and III) showed lower sequence similarities (91–96%) to known *Bifidobacterium* species, and the phylogenetic analysis shown in Figure 1A revealed that these OTUs belonged to lineages clearly distinct from known type strains. Interestingly, two of these OTUs (*Bifidobacterium* spp II and *Bifidobacterium* spp III), showed the numerically highest response to GOS (Table 1, Figure 1A).

### The population shifts induced by GOS vary among individuals

**Figure 2:**
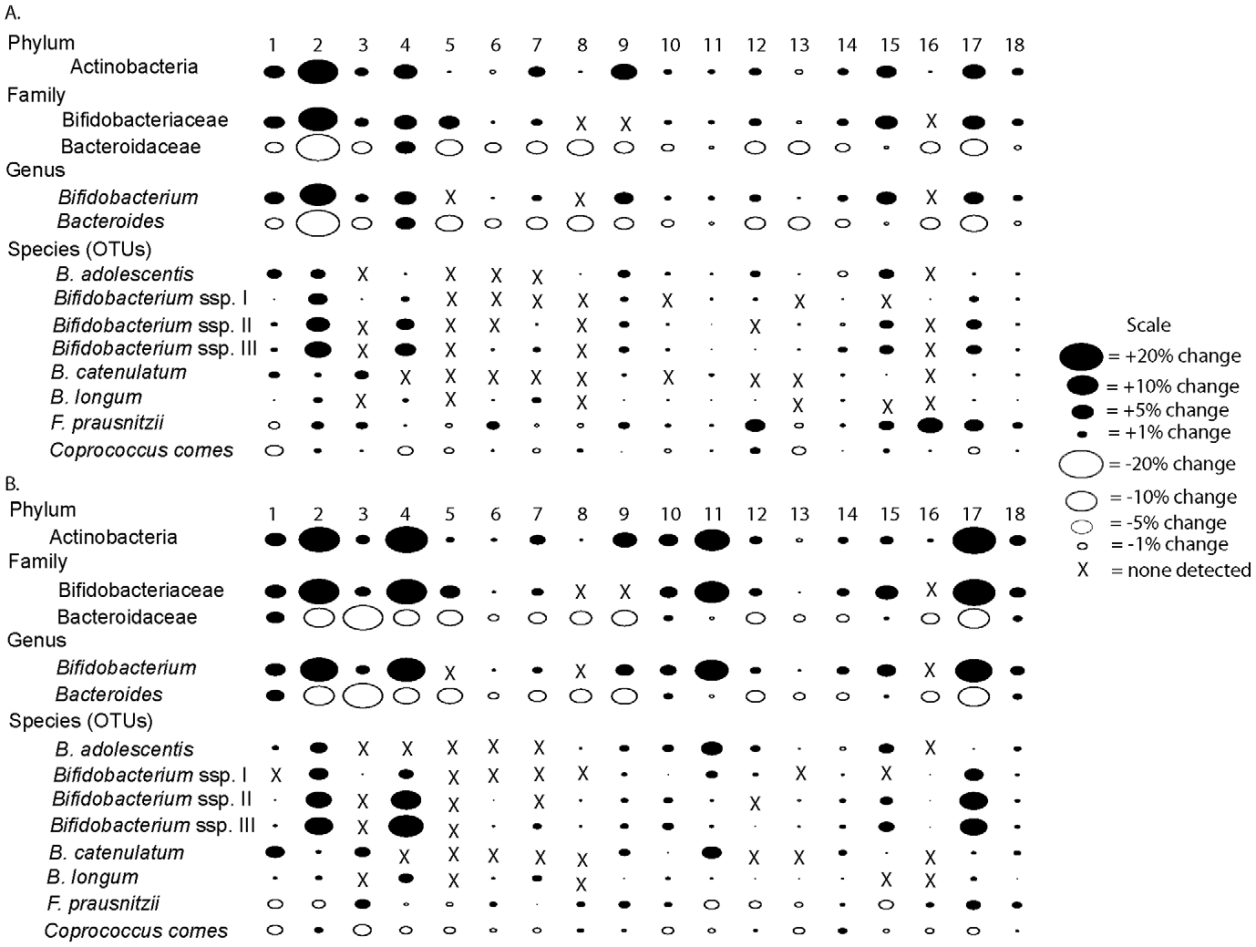
Bubble plots showing differences in the proportions of bacterial taxa as a percentage of the entire bacteria population detected during consumption of 5.0 g (A) and 10.0 g (B) when compared to the control period. The size of the bubbles is representative of the percent difference. Black ovals represent increases in proportions observed during the GOS consumption period; white ovals represent decreases.

Although the consumption of GOS at the higher doses resulted in compositional shifts within subjects on a collective basis (Figure 1B), closer examination of samples from individual subjects revealed that the effect of GOS on the intestinal composition of participants was subject to considerable variation among individuals (Figure 2). Indeed, the data showed that there were some individuals that were essentially unaffected by GOS consumption, whereas other experienced significant changes. The most substantial alteration was the increase in the Actinobacteria (at the phylum, family, genus, and species levels) which was observed in sixteen of the eighteen subjects after 5.0 g and seventeen of the subjects after 10.0 g of GOS. At the genus level, in particular, substantial increases were observed in the abundances of *Bifidobacterium*, which increased approximately ten-fold (from 1–4% up to 18–33%) in four subjects (subjects 2, 4, 11, and 17), and five-fold in seven additional subjects (subjects 1, 9, 10, 15, 18). Several culturable isolates (NEGOS 1–3) were obtained from these subjects and were found to associate within the distinct *Bifidobacterium* spp. II lineage (Figure 1A), indicating that this GOS responding linage contains bacteria that can be cultured. There was a very consistent reduction in the Bacteroidetes (at the family, genus, and species levels), which occurred within all of the subjects at some point after 5.0 g of GOS was consumed (Figure 2). At the genus level, there was a decrease in the abundance of *Bacteroides* in 17 subjects after the 5.0 g GOS dose (all except subject 4), with 14 of those subjects having a further decrease after consumption of 10.0 g of GOS.

### Temporal dynamics of microbial populations in response to GOS

**Figure 3:**
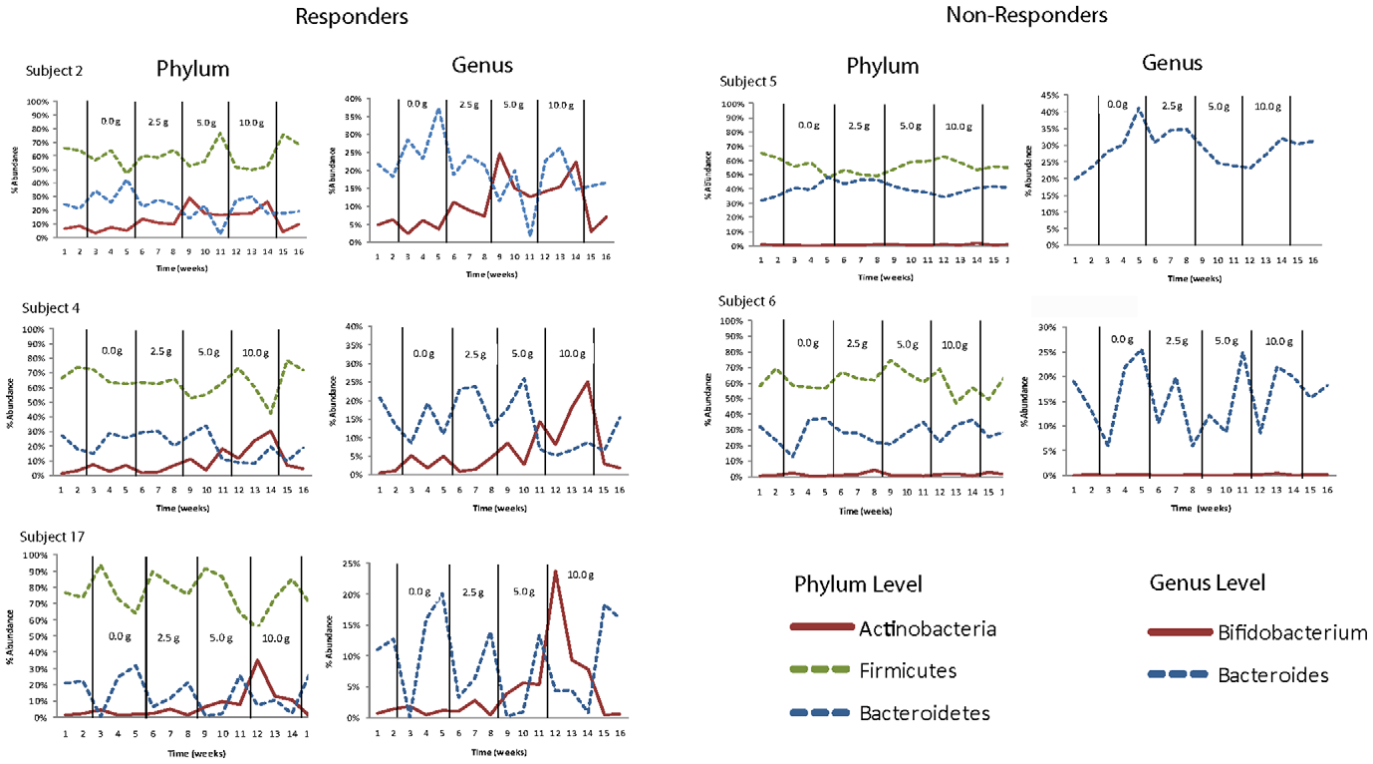
Temporal dynamics of the human fecal microbiota in response to the consumption of increasing doses of GOS shown in five human subjects. Graphs on the left show proportions of the three main phyla (Actinobacteria, Firmicutes, and Bacteroidetes) and two genera (*Bifidobacterium* and *Bacteroides*) that were affected in subjects considered as “responders”. Graphs on the right show proportions of the same three phyla and two genera for subjects considered as “non-responders”.

Analyses of the community profiles provided insight into how GOS influenced the population dynamics over the entire 16 week study period. All of the changes induced by GOS were reversible within one week, and no differences (Student's *t*-test, p>0.05) could be detected in the proportions of the bacterial groups between the first washout sample and the baseline sample (Figure 3). The temporal patterns of the three main phyla (Actinobacteria, Bacteroidetes, and Firmicutes) and two of the selected genera (*Bifidobacterium* and *Bacteroides*) for five representative subjects showed that these groups were stable in their temporal response to GOS. For example, levels of Actinobacteria, Bacteroidetes, and Firmicutes were remarkably stable in fecal samples at the baseline and washout periods, and their populations returned to the baseline level within one to two weeks after GOS consumption was stopped. The same observations were also made at the genus level for *Bifidobacterium* and *Bacteroides*.

### *In vitro* growth of gastrointestinal microbiota cultures on the prebiotic GOS

As shown above, GOS induces alterations to the human fecal microbiota that are remarkably specific for bifidobacteria. However, GOS utilization was observed to be a strain-specific phenotype, at least based on *in vitro* growth studies (Table S1). We also considered whether or not the ability to utilize GOS as a growth substrate was restricted to bifidobacteria and absent in other colonic bacteria. Therefore, we tested the ability of twenty-two strains of bacteria that are associated with the human intestinal tract to utilize GOS. This was performed by comparing growth in media containing GOS with growth in basal medium (i.e., without an additional source of carbohydrate). This experiment revealed that 6 of the 11 *Clostridium* strains could utilize GOS (Figure S3), as indicated by higher final cell densities compared to growth without carbohydrates. In addition, three of the six strains of *Bacteroides* were also significantly enriched when GOS was present. Significant growth on GOS was not observed, however, for strains of the genera *Enterococcus*, *Enterobacter*, or S*treptococcus* used in this study.

## Discussion

We recently reported that consumption of GOS induced significant bifidogenic shifts in the fecal microbial community of 18 healthy human adults [27]. Daily doses of 5.0 g were generally necessary before these effects could be observed either by cultural methods, DGGE, or qRT-PCR. In addition, we also observed that when the fecal samples from each subject were analyzed individually via DGGE or qRT-PCR, the bifidogenic response to GOS occurred consistently in only half of the subjects, whereas the other subjects were consistent “non-responders” [27]. In this current study, all 288 samples (18 subjects at 16 weekly time points) obtained during the previous study were analyzed by pyrosequencing 16S rDNA tags to obtain a community wide insight into the effects of GOS on the composition of the human fecal microbiota. The findings obtained here were entirely consistent with our previous report, but also provide new insights regarding how GOS influences the intestinal microbiota. In addition, the pyrosequencing analysis confirmed the dose-dependent bifidogenic effect of GOS. As shown in Figure 1B, the 2.5 g dose of GOS was not sufficient to induce a response, while 5 and 10 g doses were. In addition, although there was not a statistically significant difference between 5 and 10 g, there was a further increase in bifidobacteria in several subjects when the dose of GOS was increased to 10 g (Figure 1B and 2). Therefore, this study supports the suggestion made previously that there is in fact a dose response to GOS [27].

Prebiotics are described, by definition, as being “selectively fermented” and able to induce changes in the gastrointestinal microbiota that are “specific” [24]. Several previous studies have assessed the effect of GOS consumption on the stability and diversity of the human intestinal microbiota [27], [37], [44]. However, the inability to quantify the microbiota beyond the major taxa has made it difficult to test this definition and to assess the effect of prebiotics at greater resolution. The results presented here, using high throughput pyrosequencing, provide a comprehensive, high resolution analysis of the gut microbiota from individuals during a course of prebiotic consumption. The pyrosequencing results have shown, for the first time that the changes that occur during GOS consumption are remarkably restricted to a small number of bacterial groups. Indeed, the only bacteria that consistently increased in abundance in response to GOS feeding were species of bifidobacteria. Moreover, this increase in bifidobacteria abundance, to greater than 15% in some individuals, was associated with a decrease in one primary group of bacteria, namely the genus *Bacteroides* (Table 1, Figure 2). Although we also observed significant decreases in 24 OTUs within the Firmicutes phylum in several individuals (Figure S2), these differences were not significant on a subject-wide basis. Thus, we suggest that bifidobacteria enrichment by GOS occurs at the expense of a diverse collection of bacteria, including two phyla and many species. The increase, therefore, was far more specific than the decrease. Moreover, because an increase in the abundance of bifidobacteria following GOS consumption might also result in increased metabolic activity and a lower colonic pH, a broad, rather than specific inhibitory effect on the colonic microbiota would likely be expected [45].

Despite the striking selectivity of GOS, *in vivo*, microbial fermentation of GOS, *in vitro*, was much less selective, as several bacteria associated with a colonic habitat, were able to utilize GOS as a growth substrate (Figure S3). Clearly, however, as Gibson and co-workers have noted [25], the substrate preferences and competitive forces that exist within the gastrointestinal environment are quite different or absent in pure culture environments. Our findings suggest that bifidobacteria not only have the biochemical and physiological wherewithal to ferment GOS, but are also able to out-compete other members of the colonic microbiota for such specialized substrates.

Community analysis by pyrosequencing provided average sequence reads of 450 bp within the 16S rDNA gene (V1–V3 region), which was sufficient for a reliable phylogenetic assignment to the species level. Our analysis revealed that six different OTUs that belonged to the genus *Bifidobacterium* were significantly enriched through GOS. Interestingly, numerically, the most significant increase was detected for OTUs *Bifidobacterium* spp. II and III (Figure 1A). These two OTUs grouped separately from other known type strains and had only 95% and 91% homology to the type strains of *B. adolescentis* and *B. longum*, respectively. Therefore, the organisms represented by these OTUs may be distinct, as yet un-described species of GOS-responding bifidobacteria. Our data indicates that GOS enriches for different lineages within the genus *Bifidobacterium* as compared to resistant starches, which induced the abundance of bacteria that were more closely related to the type strain of *B. adolescentis* [39].

As we noted previously, the response to GOS consumption is subject to considerable individual variation [27], an observation confirmed by pyrosequencing. Of the 54 OTUs that were affected by GOS in individual subjects, 46 did not reach significance when all of the subjects were included in the analysis. In addition, none of the taxa that were significantly affected by GOS showed a response in all eighteen subjects. There are several possible explanations that may account for the highly individual response to GOS. First, non-responders might simply not harbor strains of bifidobacteria that are able to utilize GOS. Thus, the presence of specific GOS-metabolizing strains would confer responder status on that individual, whereas individuals, for whom GOS strains were absent, would be non-responders. However, when we compared the microbiota composition of the baseline samples between responders and non-responders we could not identify taxa or OTUs whose abundance was significantly lower in non-responders (data not shown). Nonetheless, it remains possible that non-responders might still lack specific strains capable of metabolizing GOS that are present in the responders. The ability of bifidobacteria to use GOS as a growth substrate is a strain specific phenotype (Table S1; [46], [47], [48]). Thus, the absence of such strains in some individuals might not be unexpected. In addition, other factors could also account for inter-subject variation, including host-specific environmental constraints, such as lumen pH or the absence of a limiting nutrient that would restrict the ability of a given bacterial group or species to increase in number even if a suitable substrate is provided [39], [45]. In addition, host digestive enzymes could, in theory, be secreted in some individuals that affect the amount of GOS that withstands digestion and reaches the colon intact. However, there is no evidence to question the non-digestibility of GOS in humans [49].

Recently, Sonnenburg and co-workers used a two-species gnotobiotic mouse model with different combinations of *Bacteroides* species to show that the impact of a prebiotic carbohydrate (inulin) on the relative abundance of the microbes could be predicted by their genetic and functional differences [5]. The authors proposed that changes in the gut microbiota brought on by dietary strategies could be inferred based on either genomic or functional knowledge of members within these populations. They further suggested that when coupled with microbiome sequence data, diet could potentially be personalized to optimize microbiota composition based on an individual's microbiota. However, the findings obtained during this study on GOS suggest that it will be difficult to predict the impact of dietary substrates on the gut microbiota. Although GOS is fermented by a wide variety of colonic bacteria *in vitro* (which obviously possess the genetic and functional attributes to ferment this substrate), it was mainly the bifidobacteria that were consistently and significantly enriched when all of the subjects were considered. Similar findings were obtained with different types of resistant starches, which only induced changes in a small number of taxa in humans although starch is widely utilized by gut bacteria [39]. We, therefore, argue that it will likely be impossible to predict the *in vivo* response of microbial communities based on metagenome sequence data of the functional attributes of individual members, without also considering the ecological and competitive interactions that occur. The latter are obviously more complex and more challenging to predict in more diverse communities than the two-species models used by Sonnenburg and coworkers [5]. To predict the impact of a dietary substrate on the gut microbiota would require more sophisticated models that take functional characteristics of the members, competitive and mutualistic interactions, and substrate preferences into account. Indeed, the prebiotic, inulin, has consistently been reported to reduce the numbers of *Bacteroides* in the human gut (probably due to a lowering of the pH) [50], [51], despite the ability of some species to ferment this substrate [52]. Therefore, we suggest that until the competitive interactions that occur in the human gut are better understood and can be integrated in predictive models, human feeding trials, such as the one described in this study, will be necessary to determine the response of dietary prebiotics on the gut microbiota.

## Supporting Information

Figure S1 **Correlation of pyrosequencing and qRT-PCR.** Pearson correlation between cell numbers and percent abundance of bifidobacteria as determined by qRT-PCR and pyrosequencing.
(TIF)

Figure S2 **Characterization of the fecal microbiota in eighteen subjects that consumed increasing doses of GOS by multiplex pyrosequencing of 16S rDNA tags.** Phylogenetic trees that encompass the phyla, Firmicutes (**A**) and Bacteroidetes (**B**) are shown. The trees contain representative sequences of all OTUs that were significantly affected by GOS in individual subjects together with sequences of related entries in the database (which included both type strains of known species and sequences from molecular studies of human fecal samples). Sequences were aligned in Muscle 3.6 and the trees were built using the neighbor-joining algorithm with 1,000 bootstrap replicates in MEGA 4.0. Open black, closed black, and grey symbols were used to label sequences from individual subjects. OTUs that were not significantly affected in any of the eighteen subjects were labeled as “NS”. Arrows to the right of each cluster indicate the number of subjects that showed statistical significance after ANOVA analysis. The direction of the arrow indicates either a significant increase (↑) or significant decrease (↓) for each subject showing significance for that particular OTU cluster.
(TIF)

Figure S3 **Twenty-two anaerobic bacteria of human gastrointestinal origin were screened** ***in vitro*** **to determine their ability to utilize GOS.** Average optical densities and standard deviations for each of the strains are shown, with GOS-grown cultures in shaded bars and control cultures in open bars. Significant differences were determined by students T-test and indicated by asterisks, where p<0.05.
(TIF)

Table S1 **Growth of bifidobacteria on galactooligosccharides.**
(DOC)
