# Supplementary figures and images for: Barcoded Pyrosequencing Reveals That Consumption of Galactooligosaccharides Results in a Highly Specific Bifidogenic Response in Humans
Source: PLoS One. 2011 Sep 26;6(9):e25200. doi: 10.1371/journal.pone.0025200 (PMC3180383; doi:10.1371/journal.pone.0025200)

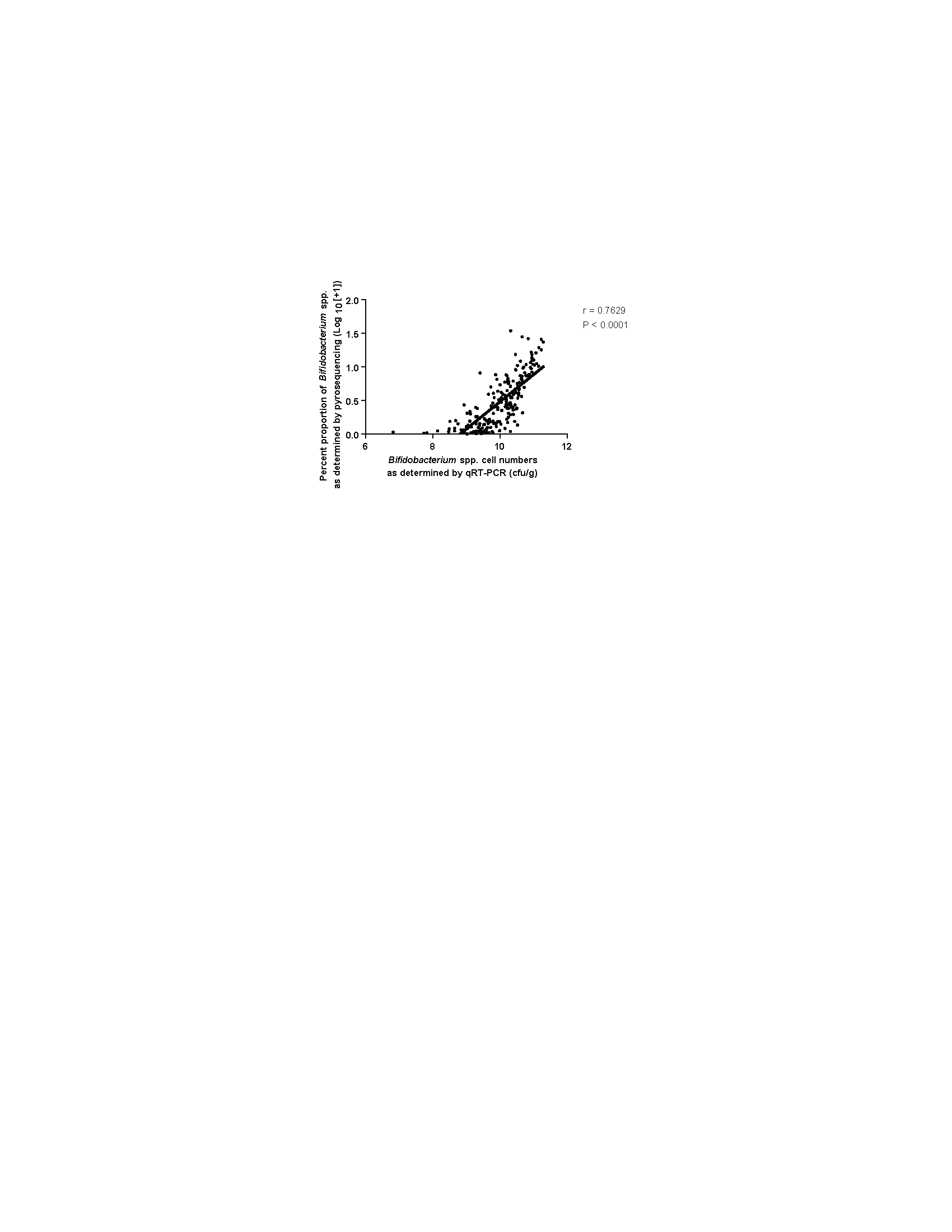

Supplement: Figure S1 — Correlation of pyrosequencing and qRT-PCR. Pearson correlation between cell numbers and percent abundance of bifidobacteria as determined by qRT-PCR and pyrosequencing. (TIF) [file pone.0025200.s001.tif]

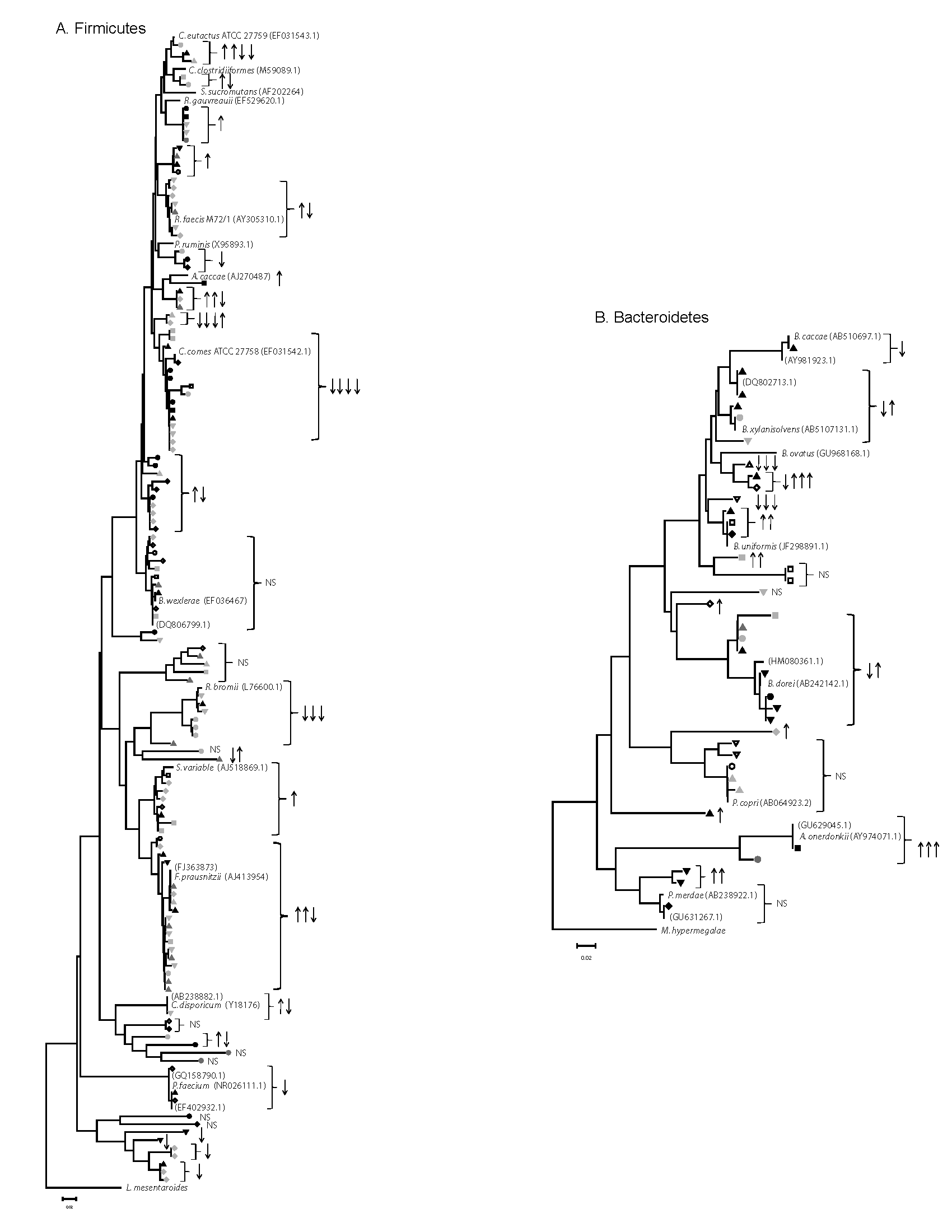

Supplement: Figure S2 — Characterization of the fecal microbiota in eighteen subjects that consumed increasing doses of GOS by multiplex pyrosequencing of 16S rDNA tags. Phylogenetic trees that encompass the phyla, Firmicutes (A) and Bacteroidetes (B) are shown. The trees contain representative sequences of all OTUs that were significantly affected by GOS in individual subjects together with sequences of related entries in the database (which included both type strains of known species and sequences from molecular studies of human fecal samples). Sequences were aligned in Muscle 3.6 and the trees were built using the neighbor-joining algorithm with 1,000 bootstrap replicates in MEGA 4.0. Open black, closed black, and grey symbols were used to label sequences from individual subjects. OTUs that were not significantly affected in any of the eighteen subjects were labeled as “NS”. Arrows to the right of each cluster indicate the number of subjects that showed statistical significance after ANOVA analysis. The direction of the arrow indicates either a significant increase (↑) or significant decrease (↓) for each subject showing significance for that particular OTU cluster. (TIF) [file pone.0025200.s002.tif]
