# Supplementary material for: Barcoded Pyrosequencing Reveals That Consumption of Galactooligosaccharides Results in a Highly Specific Bifidogenic Response in Humans
Source: PLoS One. 2011 Sep 26;6(9):e25200. doi: 10.1371/journal.pone.0025200 (PMC3180383; doi:10.1371/journal.pone.0025200)
Supplement: Table S1 — Growth of bifidobacteria on galactooligosccharides. (DOC) [file pone.0025200.s004.doc]

| Species (total number) | Number positive (%) | Number negative (%) |
| --- | --- | --- |
| *B. adolescentis* (10) | 6 (60%) | 4 (40%) |
| *B. bifidum* (6) | 5 (84%) | 1 (16%) |
| *B. breve* (2) | 1 (50%) | 1 (50%) |
| *B. animalis* subsp. *lactis* (1) | 1 (100%) | 0 (0%) |
| *B. longum* subsp. i*nfantis* (2) | 2 (100%) | 0 (0%) |
| *B. longum* subsp *longum* (9) | 6 (67%) | 3 (33%) |
| *B. pseudocatenulatum* (3) | 2 (67%) | 1 (33%) |
| Other *Bifidobacterium* spp. (6) | 3 (50%) | 3 (50%) |
| Total (39) | 26 (67%) | 13 (33%) |

Cultures were considered positive when the OD at 24 h was > 0.4 higher than control cultures
